# Supplementary material for: Genetic Alterations in Mitochondrial DNA Are Complementary to Nuclear DNA Mutations in Pheochromocytomas
Source: Cancers (Basel). 2022 Jan 6;14(2):269. doi: 10.3390/cancers14020269 (PMC8773562; doi:10.3390/cancers14020269)
Supplement: Supplementary file 1 [file cancers-14-00269-s001.zip › cancers-1477543-supplementary.pdf]

cancers

MDPI

Genetic Alterations in Mitochondrial DNA Are Complementary to Nuclear DNA Mutations in Pheochromocytomas

Mouna Tabebi, Małgorzata Łysiak, Ravi Kumar Dutta, Sandra Lomazzi, Maria V. Turkina, Laurent Brunaud, Oliver Gimm and Peter Söderkvist

Table S1. Clinical information of patients with pheochromocytomas and paragangliomas from Nancy, France and from Linköping, Sweden.

Case ID	Age	Gender	Tumor size [mm]	Malignancy
		French cohort		
F3	78	M	70	Benign
F4	29	M	10	Benign
F5	57	F	30	Benign
F6	31	F	20	Benign
F7	58	M	25	Benign
F8	60	M	35	Benign
F9	37	F	100	Benign
F10	70	M	40	Benign
F11	23	M	40	Benign
F12	30	M	35	Benign
F14	70	F	37	Benign
F15	52	F	20	Benign
F16	75	F	70	Benign
F17	40	F	40	Benign
F18	57	M	25	Benign
F19	66	M	27	Benign
F20	46	M	37	Benign
F21	83	M	50	Benign
F23	34	M	20	Benign
F24	50	M	15	Benign
F26	62	F	50	Benign
F27	61	M	27	Benign
F28	64	M	55	Benign
F29	83	M	50	Benign
F30	65	F	14	Benign
F31	48	F	60	Benign
F32	64	F	47	Benign
F33	66	M	21	Benign
F34	59	M	30	Benign
F35	80	M	90	Benign
F36	40	M	30	Benign
F37	66	F	40	Benign
F38	69	M	30	Benign
F39	55	F	90	Benign
F40	44	F	30	Benign
F41	58	F	50	Benign
F42	62	F	50	Benign
F43	51	F	30	Benign
F44	41	M	33	Benign
F45	45	F	38	Benign
F46	27	F	40	Benign

F47	24	F	45	Benign
F48	49	F	40	Benign
F49	67	F	50	Benign
F51	52	F	25	Benign
F52	36	F	45	Benign
F53	50	F	25	Benign
F54	52	F	50	Benign
F55	72	M	40	Benign
F56	30	M	65	Benign
F57	63	M	40	Benign
F58	44	M	50	Benign
F59	61	F	45	Benign
F60	46	F	11	Benign
		Swedish cohort		
PH34	54	M	17	Benign
PH 35	75	M	60	Benign
PH 36	71	F	35	Benign
PH 37	70	F	30	Benign
PH 38	47	M	30	Benign
PH 40	63	F	32	Benign
PH 41	68	F	20	Benign
PH 42	62	F	30	Benign
PH 44	68	F	55	Benign
PH 45	58	M	30	Benign
PH 57	58	M	30	Benign
PH 58	48	F	35	Benign
PH 60	78	M	80	Benign
PH 61	63	F	100	Liver infiltration, patient died after 7 months
PH 62	33	M	26	Benign
PH 63	69	M	35	Benign
PH 64	56	M	32	Benign
PH 65	17	F	15	Benign
PH 66	26	F	50	Benign
PH 67	38	M	55	Benign
PH 68	66	F	50	Benign
PH 79	65	F	70	Benign
PH 80	56	M	36	Benign

F, Female; M, male. The cohort included 74 pheochromocytomas, and three paragangliomas (F7, F27, F43); FR7: retrocaval paraganglioma at the level of left renal vein = abdominal; FR27: paraganglioma near the left adrenal gland (without continuity with adrenal gland) = abdominal; FR43: paraganglioma next to the right adrenal gland = abdominal.

Table S2. List of genes selected for the gene panel sequencing.

Gene name	Type of mutation	Molecular pathway	References
	**Susceptibility genes**		
	Very common (>10%)		
EPAS1/HIF2A	Mosaic or somatic	Pseudohypoxia signaling	[18]
SDHB	Germline	Pseudohypoxia signaling	[19]
VHL	Germline or somatic	Pseudohypoxia signaling	[19]
NF1	Germline or somatic	MAPK pathway	[20]
HRAS	Somatic	MAPK pathway	[21]
RET	Germline or somatic	MAPK pathway	[21]
SDHD	Germline	Pseudohypoxia signaling	[21]
	Common (5%-10%)		
MET	Germline or somatic	MAPK pathway	[22]
Uncommon (<5%)			

ATRX	Somatic	Chromatin remodeling	[22]
FGFR1	Somatic	MAPK pathway	[23]
KIFB1B	Germline or somatic	Hypoxia-independent pathway	[23]
Rare (2%)			
BRAF	Somatic	Pseudohypoxia signaling	[21]
FH	Germline	Pseudohypoxia signaling	[22]
MAX	Germline or somatic	MYC pathway	[19]
SDHA	Germline	Pseudohypoxia signaling	[24]
SDHC	Germline	Pseudohypoxia signaling	[21]
TMEM127	Germline	mTOR negative regulator	[25]
Very rare (1%)			
EGLN1/PHD2	Germline or somatic	Pseudohypoxia signaling	[26]
MYCN	Somatic	MYC pathway	[27]
SDHAF2	Germline or somatic	Pseudohypoxia signaling	[28]
**Candidate genes**			
BRAF	somatic	RAS/RAF/ERK signalling	[29]
SCAI	LOH	Wnt/β-catenin signaling	Unpublished data
BAP1	Germline	AMPK-mTOR signalling	[30]
CSDE1	Somatic	Wnt signaling	[1]
D2HGDH	Germline	Pseudohypoxia signaling	Unpublished data
UBTF1	Fusion	MAPK pathway	[1]
TCF4	Fusion	Wnt signaling	[1]

Table S3. cDNA and DNA primers used for nuclear genes amplification, Sanger sequencing and RT-qPCR.

Target	Forward / Reverse primer
**HRAS mutation analysis**	
Exon 2F	5′-GTGGGTTTGCCCTTCAGAT-3′
Exon 2R	5′-CGCCAGGCTCACCTCTAT-3′
Exon 3F	5′-ATTCCTACCGGAAGCAGGTGG-3′
Exon3R	5′-CTCACGGGGTTCACCTGTACTG-3′
**EGLN mutation analysis**	
Exon 1-1F	5′-GACCGGCAGTACTGCGA-3′
Exon 1-1FR	5′-GCTTGGCCTTTACTTTTCCCT -3′
Exon 1-2F	5′-TTCCAGGAGAAGGCGAACC -3′
Exon 1-2R	5′-CTTATCGCCTCGGATGTCCT -3′
**TFAM mutation analysis**	
TFAM SEG1 F	5′-GCTGGAGTTGTGTATTGCCA-3′
TFAM SEG1 R	5′-TATATACCTGCCACTCCGCC-3′
TFAM SEG2 F	5′-AGAAGAATTGCCCAGCGTTG-3′
TFAM SEG2 R	5′-TGGTTTCCTGTGCCTATCCA-3′
**POLG1 mutation analysis**	
POLG1 SEG1 F	5′-CAGGACGTGTCTCTCTCCAC-3′
POLG1 SEG1 R	5′-AGTAACGCTCTTCCACCAGC-3′
POLG1 SEG2 F	5′-ACGTGGAGGTCTGCTTGG-3′
POLG1 SEG2 R	5′-CATGCCGGCCAGAGTCAC-3′
POLG1 SEG3 F	5′-AGGACCTGATGCAGTACTGT-3′
POLG1 SEG3 R	5′-CTGCTTCCCCTGTTCGAGA-3′
POLG1 SEG4 F	5′-GAGTCAGCTGGGGTGGTC-3′
POLG1 SEG4 R	5′-TCCTCATCATAGTCGGGGTG-3′
POLG1 SEG5 F	5′-CTGCCCAGGTCAGCTCTG-3′
POLG1 SEG5 R	5′-CCTCCCACTTCTTCCACTGT-3′
POLG1 SEG6 F	5′-CGCAAGGTCCAGAGAGAAAC-3′
POLG1 SEG6 R	5′-CACGGGAGCAAATACAGAGC-3′
Exon 21F	5′- TTCCCAGTTTATGACCAGCC-3′

Exon 21R	5′- AAGGAACGCTCACCCAAAG-3′
Exon 23F	5′-TGGCATCCTAACCAATTTGC-3′
Exon 23R	5′- GAAAGTGGGGAAAGCATGAC-3′
**C10ORF2 mutation analysis**	
PEO1 SEG1 F	5′-ACGCTAACCAGGCACCTAAG-3′
PEO1 SEG1 R	5′-AAGGGAAGACAAGACTGCGA-3′
PEO1 SEG2 F	5′-TCGCAGTCTTGTCTTCCCTT-3′
PEO1 SEG2 R	5′-GTCTTTCCACTGCCTGTTGG-3′
PEO1 SEG3 F	5′-GAAAGGGCGAGCTGACGG-3′
PEO1 SEG3 R	5′-TGATGTAGTCTTGAGCTGCGA-3′
PEO1 SEG4 F	5′-ACCTGCAGTTCATGATGGGT-3′
PEO1 SEG4 R	5′-GACCACAGGATAGAGGAGCC-3′
**DGUOK mutation analysis**	
Exon 1F	5′-TTACGTCAACGGTGCGCTGGAGCGA-3′
Exon 1R	5′-CGGCCGCATCAGACGCAGGCCATTC-3′
Exon 2F	5′-CAATGGTACGGCTGCTGAGT-3′
Exon 2R	5′-TGGTGTATGTGGTCTGCTTTC-3′
Exon 3F	5′-TAAACCTGTTTGGGGAGGTAG-3′
Exon 3R	5′-CTCTGGACAAATCTGTATAGT-3′
Exon 4F	5′-GGTTAAGTGGTTTGAACAGA-3′
Exon 4R	5′-TGTGCTGCAGGTAATGTCTAC-3′
Exon 5F	5′-GCAAAGGCATGGCTTGTAATG-3′
Exon 5R	5′- TGACATTTCCAACCATTTCCAG-3′
Exon 6F	5′-AGATCTGTTCTCTGAGTAAGAC-3′
Exon 6R	5′-GTGTGAAATGCAATGTGAGATG-3′
Exon 7F	5′-TGCCCACATTTCTTCTCCATG-3′
Exon 7R	5′-CAGCTCTTGAGCGGCAGAG-3
**MPV17 mutation analysis**	
Exon 1F	5′-TGCCCCTTTAAGTCACATTG-3′
Exon 1R	5′-CTTCCAAATCAGTCTGCC-3'
Exon 2F	5′-AATGGCAGCCCATCCCAGGA-3′
Exon 2R	5′-GTCCAAGGGAAGCCAAAGGA-3′
Exon 3+4F	5′-AGCTTAGTGAGGTAGAGGC-3'
Exon 3+4R	5′-AAACCTGTCTTCTTCCCC-3'
Exon 5+6+7F	5′-AGGAGAACAGAGTGGGGA-3'
Exon 5+6+7R	5′-TGTTGGTAACGTGGGTCTT-3'
Exon 8F	5′-GCCATTTCATGCTCCCAT-3′
Exon 8R	5′-CTCTAGAAATGACTCCCC-3′
**6mA mitochondrial methylation**	
6mA-1 F	5′-CCTCCCACTCCCATACTACTAATCTC-3′
6mA-1 R	5′-GTCTTTGGGGTTTGGTTGGTT-3′
6mA-2 F	5′-CAATAGCCTCATCATCCCCACC-3′
6mA-2 R	5′TACGTTGTTAGATATGGGGAGTAGTGT3′
6mA-6 F	5′-TCATACTCTTTCACCCACAGCACC-3′
6mA-6 R	5′-CTACAGCGATGGCTATTGAGGAG-3′
6mA negative-2 F	5′-ACATCGGCATTATCCTCCTGC-3′
6mA negative-2 R	5′-ACTGTGGCCCCTCAGAAT-3′
**Gene expression (RT-qPCR)**	
TFAM F	GAACAACTACCCATATTTAAAGCTCA
TFAMR	GAATCAGGAAGTTCCCTCCA
PGC-1A F:	TGAGAGGGCCAAGCAAAG
PGC-1A R:	ATAAATCACACGGCGCTCTT
NRF1 F:	AGGAACACGGAGTGACCCAA
NRF1 R:	TATGCTCGGTGTAAGTAGCCA
LC3A F:	CATGAGCGAGTTGGTCAAGAT
LC3A R:	TCGTCTTTCTCCTGCTCGTAG

P62 F:	GGGGACTTGGTTGCCTTTT
P62 R:	CAGCCATCGCAGATCACATT
GUSB F:	CAAGACAGTGGGCTGGTGAATTA
GUSB R:	CTTGAACAGGTTACTGCCCTTGAC

Table S4. Statistics of the GSEA of Swedish tumors compared to French tumors. Enrichment pattern with FDR lower than 25% was considered significant.

GSEA PATHWAY	NES	p-val	FDR
**Down-regulated gene sets**			
REACTOME_METABOLISM_OF_PROTEINS	-2.27	0.000	0.002
REACTOME_TRANSLATION	-2.2	0.000	0.002
REACTOME_3_UTR_MEDIATED_TRANSLATIONAL_REGULATION	-2.13	0.000	0.004
REACTOME_PEPTIDE_CHAIN_ELONGATION	-2.12	0.000	0.006
KEGG_RIBOSOME	-2.09	0.002	0.007
REACTOME_NONSENSE_MEDIATED_DECAY_ENHANCED_BY_THE_EXON_JUNCTION_COMPLEX	-2.04	0.002	0.01
REACTOME_SRP_DEPENDENT_COTRANSLATIONAL_PROTEIN_TARGETING_TO_MEMBRANE	-2.03	0.000	0.01
REACTOME_INFLUENZA_VIRAL_RNA_TRANSCRIPTION_AND_REPLICATION	-1.97	0.002	0.023
REACTOME_INFLUENZA_LIFE_CYCLE	-1.91	0.004	0.044
REACTOME_METABOLISM_OF_MRNA	-1.9	0.003	0.048
REACTOME_ACTIVATION_OF_THE_MRNA_UPON_BINDING_OF_THE_CAP_BINDING_COMPLEX_AND_EIFS_AND_SUBSEQUENT_BINDING_TO_43S	-1.88	0.005	0.054
REACTOME_METABOLISM_OF_RNA	-1.85	0.004	0.065
**Up-regulated gene sets**			
REACTOME_GPCR_DOWNSTREAM_SIGNALING	2.34	0.000	0.000
REACTOME_SIGNALING_BY_GPCR	2.15	0.000	0.004

NES: normalized enrichment score.

Table S5. Association between mtDNA haplogroups bins and PCCs/PGLs.

Macro-haplogroups	Controls N=1888	PCCs and PGLs N=77	OR	95%CI	p-value
**HV/H/V**	936 (49.6%)	41 (53.5%)	1		
**J/T**	349 (18.5%)	7 (9.5%)	0.458	[0.203-1.03]	0.053
**U/K**	425 (22.5%)	15 (19%)	0.806	[0.441-1.472]	0.479
**Other haplogroups***	178 (9.4%)	14 (18%)	1.796	[0.959-3.363]	0.064

* Rare haplogroups in our studied cohorts; CI: confidence interval; mtDNA: mitochondrial DNA; PCCs: pheochromocytoma; PGLs: paragangliomas; OR: odds ratio.

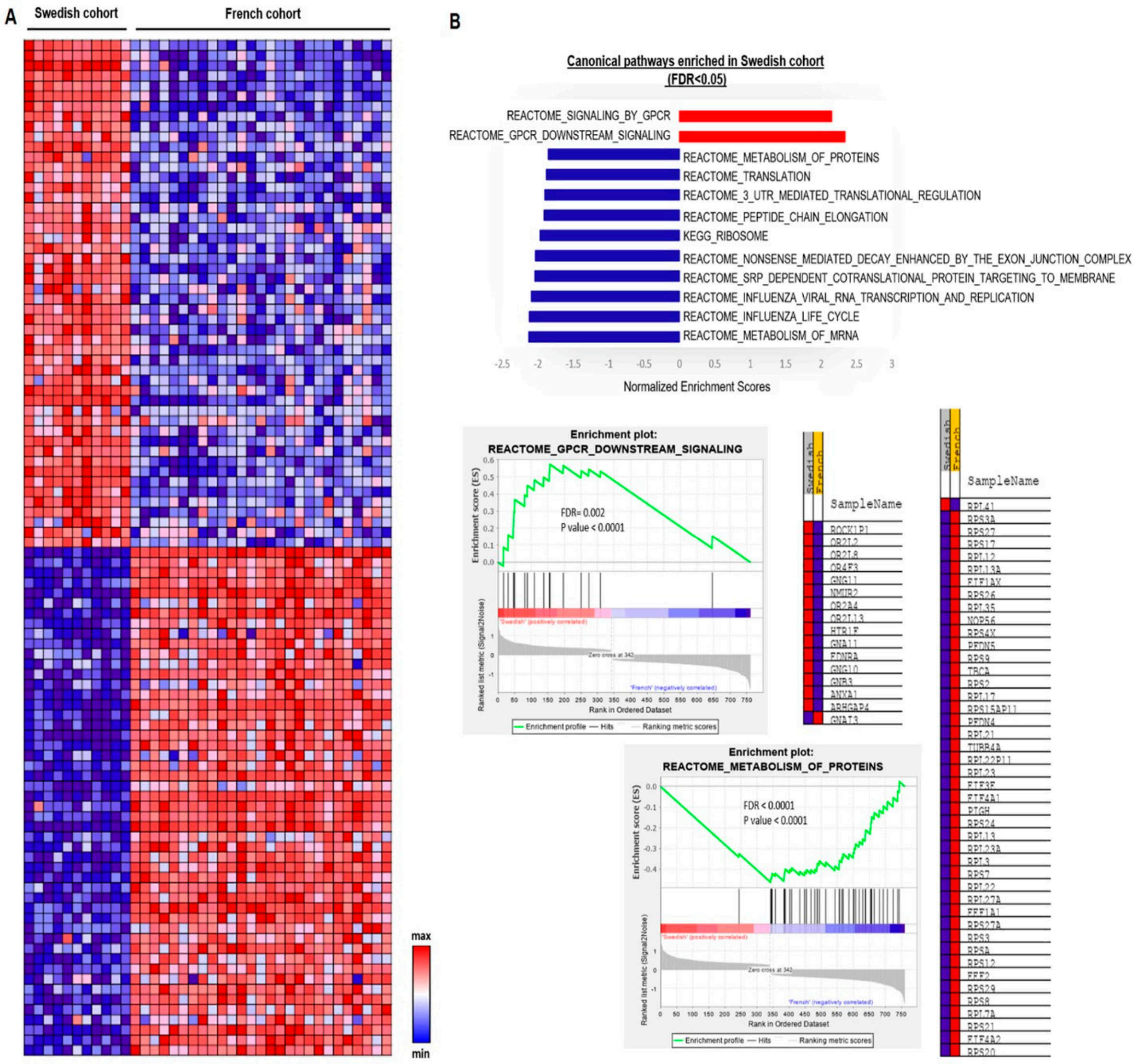

Figure S1. A) Heatmap with top 50 differentially expressed genes, in Swedish and French PCCs/PGLs, after Gene set enrichment analysis (GSEA). B) GSEA enrichment plots of canonical pathways in Swedish tumors: Reactome metabolism of proteins and Reactome GPCR downstream signaling.

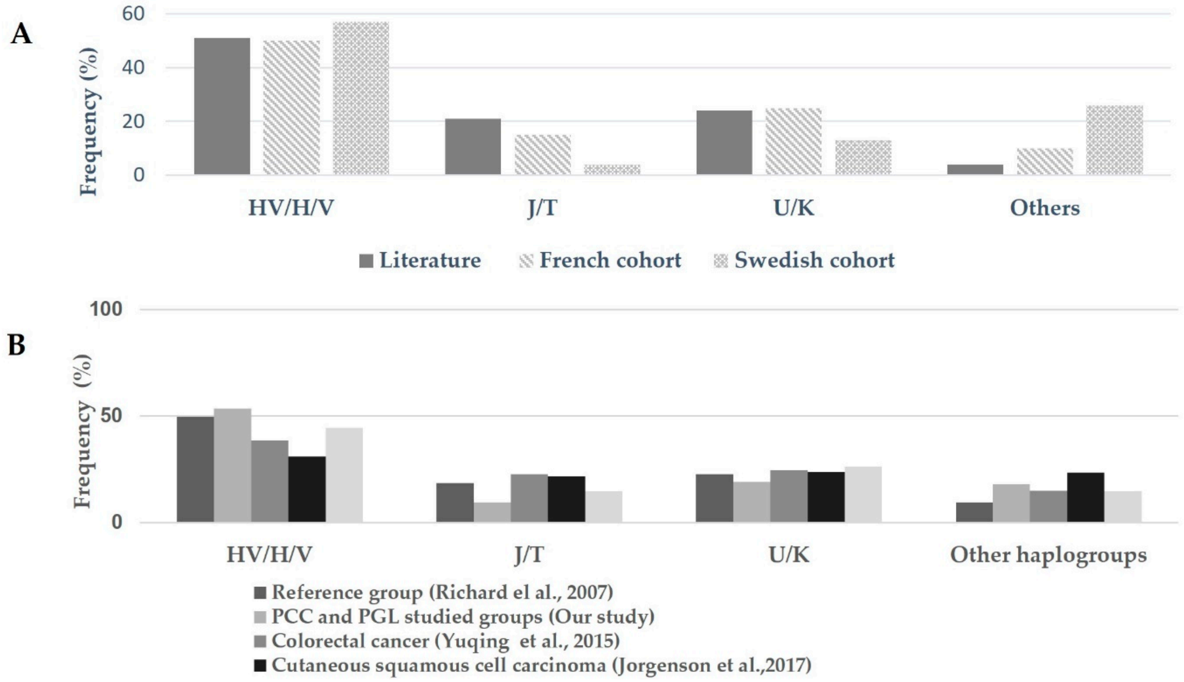

Figure S2. Haplogroup distribution in all studied PCCs/PGLs tumors (our study) (A) compared with a healthy control cohort (Reference group) and other types of cancer (B).

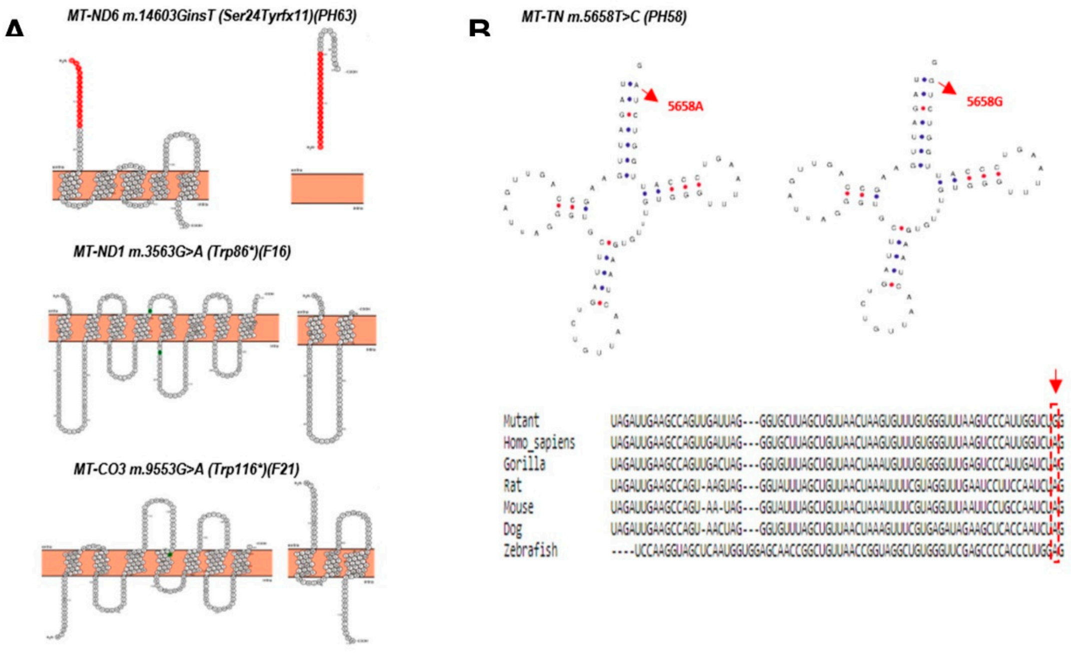

Figure S3. A) Predicted transmembrane structures of the human wild-type ND6, ND1 and CO3 proteins (left) and in presence of the novel truncating mutations (right). B) Schematic secondary structure of the human MT-TN gene showing marked with red the novel mutation described in our study. Nucleotide sequence alignment of human MT-TN gene through different species showing that the A nucleotide in position 72 (red arrow) is conserved among species.

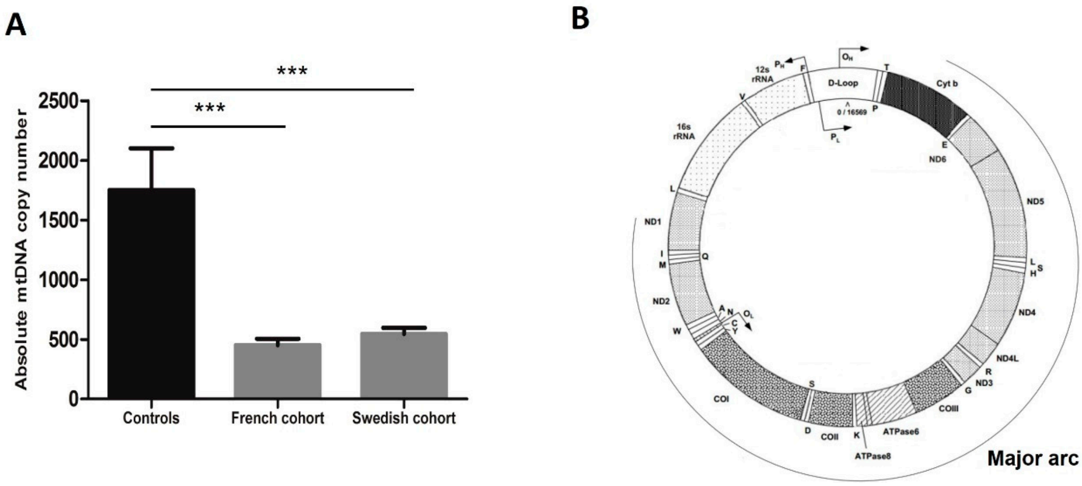

Figure S4. (A) Absolute mtDNA copy number in the Swedish tumors, French tumors, and controls (normal adrenal medulla tissues); *p*-values were calculated by *t*-test, *** <0.0001. (B) Morbid Map of the Human mtDNA Genome (www.mitomap.org) showing the major arc.

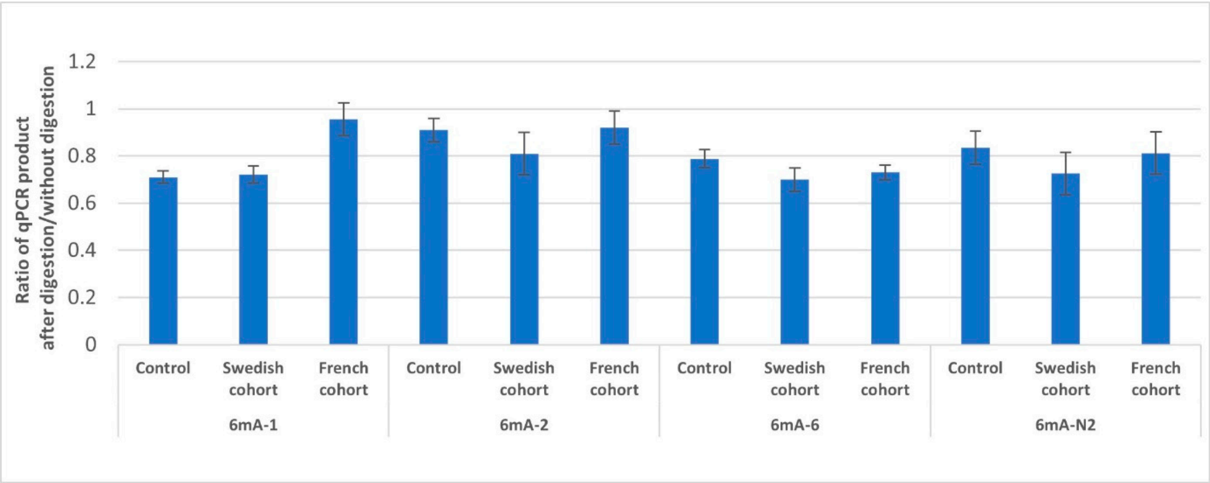

Figure S5. Semi-qPCR results of 4 selected CATG sites. After DpnI digestion, semi-qPCR was performed using specific primers covering these sites. The ratio was calculated between the PCR product concentration from digested and undigested DNA samples (mean ± standard error).
